# Risk of cervical intraepithelial neoplasia grade 3 or higher (CIN3+) among women with HPV-test in 1990–1992, a 30-year follow-up study

**DOI:** 10.1186/s13027-021-00386-z

**Published:** 2021-06-22

**Authors:** Marit Østlyngen Riibe, Sveinung Wergeland Sørbye, Gunnar Skov Simonsen, Arnfinn Sundsfjord, Josef Ekgren, Jan Martin Maltau

**Affiliations:** 1 Department of Medical Biology, UiT The Arctic University of Norway, Tromsø, Norway; 2 Department of Clinical Pathology, University Hospital of North Norway, Tromsø, Norway; 3 Department of Microbiology and Infection Control, University Hospital of North Norway, Tromsø, Norway; 4 Department of Obstetrics and Gynaecology, University Hospital of North Norway, Tromsø, Norway

**Keywords:** Cervical Cancer, Human papillomavirus, Cervical intraepithelial neoplasia grade 3 or worse, Long-term follow-up

## Abstract

**Background/objective:**

Having a 30-year follow-up of a cohort of women tested for HPV is a unique opportunity to further study long-term risk of CIN3+. The study objective was to compare HPV status at baseline with the risk of CIN3+ in the follow-up period of 30 years.

**Methods:**

All women (*n* = 642) referred to the HPV outpatient clinic at the University Hospital of North Norway (UNN) in 1990–1992, with an HPV test at baseline, were included in a prospective cohort. HPV-testing was performed by two different HPV-DNA tests, and genotypes 6, 11, 16, 18, 31 and 33 were identified. High-risk (HR) HPV genotypes (16, 18, 31 and 33) were classified as HPV positive, whereas low-risk (LR) genotypes (6 and 11) in addition to absent HPV were classified as HPV negative. A single cohort in which women were classified for their HPV status underwent follow-up prospectively to the last time-point of observation of 30 years.

**Results:**

During follow-up, 148 (148/642) cases of CIN3+ were detected, of whom 70.3% (104/148) were HPV positive and 29.7% (44/148) were HPV negative at baseline. The proportions of women who developed CIN3+ following a positive and a negative test were 46.6% (104/223) and 10.5% (44/419), respectively. Most cases of CIN3+ were seen shortly after the baseline HPV test, with 112 cases of CIN3+ diagnosed within the first year. In total, 48.6% (72/148) with HPV 16 and 57.6% (19/33) with HPV 33 developed CIN3+. Within the first year, CIN3+ was detected in 37.8% (56/148) with HPV 16, and 51.5% (17/33) with HPV 33. The long-term risk of CIN3+ was significantly lower than the short-term risk, and mainly associated with HPV 16. Overall, eight cases of cervical cancer were detected. Five were HPV positive, harboured HPV 16 at baseline and developed cervical cancer after 3, 4, 5, 11 and 24 years of follow-up.

**Conclusion and consequences:**

HPV status at baseline is predictive for the subsequent risk of developing CIN3+. Women with a positive HPV test in 1990–1992 had a significantly higher risk of CIN3+ during 30 years of follow-up than those with a negative test. HPV 16 was associated with the greatest long-term risk of cervical cancer. All patients with a positive HPV test at baseline should be followed up until negative.

**Trial registration:**

ISRCTN, ISRCTN10836802. Registered 14 December 2020 - Retrospectively registered.

**Supplementary Information:**

The online version contains supplementary material available at 10.1186/s13027-021-00386-z.

## Introduction

The importance of Human Papilloma Virus (HPV) in the evolution of cervical intraepithelial neoplasia (CIN) and cancer is well-established [1, 2]. Persistent infections with high-risk (HR) HPV are the main cause, and 84.3% of cervical cancers are attributable to one of the five most common HR-HPV types (16, 18, 45, 33, 31) [3]. Although HPV infection is the most important cause of cervical cancer, it is very common and usually transient [2, 4]. Within the general screening population, approximately 30% of those under the age of 30, and 6–7% between 34 and 69 years, have an ongoing HPV infection with one or more of the 14 most common HR-genotypes [5]. A study of adolescent and young women in the US found that approximately 70% with a positive HPV test will render negative within 24 months [6]. It has been estimated that the mean calculated time from infection to diagnosis of CIN3+ is 9.4 years (SD 4.1 years) and progression from CIN3 to invasive cervical cancer takes 10–20 years, depending on genotype [7, 8]. A recent modelling study by Burger et al. found that the median time from acquisition of HPV to cancer detection ranged from 17.5 years to 26.0 years [9].

The Norwegian Cervical Cancer Screening Program (NCCSP), established in 1995, recommended primary screening by cervical cytology in the age group 25–69 years [10]. Traditionally, conventional cytology has been the method of choice. Today, liquid-based cytology is preferred due to the possibility of performing an HPV test in the same sample. HPV screening is recommended by almost all guidelines in the world. A higher sensitivity and higher effectiveness in detecting clinically important CIN3 [11–15] and in preventing cancer than cytology [16] was demonstrated in the first decade of 2000 and is the evidence behind the actual recommendations. Based on this, the NCCSP has now been revise to include both cytology-based and HPV-based primary screening. Women aged 25–33 years are recommended cytology-based screening every third year and women 34–69 years are recommended HPV-test every 5 years. HPV-test in triage of women with minor cervical lesions was implemented in 2005.

A single positive test for HR-HPV in women with normal cytology is predictive of her subsequent risk for developing CIN2+ [17–19]. Having a 30-year follow-up of a cohort of women tested for HPV is a unique opportunity to further study long term risk of CIN3+. In 1990, an HPV outpatient clinic was established at UNN Tromsø as a collaboration between specialists in different departments, including gynaecologists, pathologists and microbiologists. At that time the NCCSP did not exist, but screening by cervical cytology was well known. The HPV clinic was established to study the presence and diversity of HPV in an outpatient clinic more closely. The tests used detected four of the five most common HR-HPV types in cervical cancer including 16, 18, 31 and 33. They also detected LR-HPV 6 and 11, associated with genital condylomas. We compared HPV status at baseline with the risk of CIN3+ in the follow-up period of 30 years.

## Methods

### Study population

From July 1st 1990 to October 15th 1992, all women referred to the HPV outpatient clinic at UNN Tromsø, having an HPV test at baseline, were included in a prospective study. They were referred from their primary care physician, mainly within Troms and Finnmark counties. The reason for referral was symptoms associated with genital condylomas, cervical- and vulvar dysplasia, resistant vaginal discharge, vulvodynia, vulvar lichen planus or pruritus. Six hundred forty-two women were eligible for study participation. Age at inclusion was between 16 and 70 years. A clinical and colposcopic examination was performed in all patients. Paired samples for HPV testing were taken from cervix and vulva (cytology and biopsy) and visible lesions. The cervical scrapings and biopsy for HPV testing were taken from the endocervical canal and the transformation zone on the ectocervix. Patients with a histological confirmed CIN2+ were offered treatment. HPV results were not used to direct patient management.

### HPV DNA testing

HPV-testing was performed by a two-step nonradioactive Southern blot DNA hybridization method using biotinylated HPV-specific probes (ONCOR, Medscan AB PO Box 20,047 S-100 74 Malmø, Sweden). HPV-types identified were 6, 11, 16, 18, 31 and 33 causing 78.3% of all cervical cancer [3]. In addition, a polymerase chain reaction (PCR) method using degenerate L1 consensus primers (MY09/MY11) was performed on samples shown to be adequate for PCR amplification by separate β-globulin PCR with PCO4 and GH20 primers as described [20].

PCR amplifications were done in a GeneAmp PCR System 9600 (Perkin Elmer Cetus, Emeryville, CA), with the following modifications. PCR reaction condition with each primer at 0.25 μM of each primer, 100 μM of each deoxynucleotide and 1 U Taq polymerase in a final reaction volume of 50 μl were used. To obtain reproducible HPV PCR-results it was necessary to use hot-start PCR to avoid primer-dimer artefacts, adding Taq-polymerase after heating the samples at 80 °C for 2 min. The cycling conditions were 1 min at 94 °C, 30 s at 55 °C, 30 s at 72 °C; and a final three-minute extension interval at 72 °C. An aliquot of 15 μl of the PCR-products was analysed on ethidium bromide-stained 2% agarose gels. All experiments were done in parallel with positive (diluted cloned HPV-DNAs) and negative controls (ddH_2_O). PCR-products were further identified with separate HPV 6, 11, 16, 18, 31 and 33 dot blot hybridizations using specific alkaline phosphatase-labelled oligonucleotides as earlier described [20]. HPV amplicons not identified by any of these oligoprobes were not further examined. The test result was defined as positive when HPV was identified in one or both methods. HR-HPV genotypes (16, 18, 31 and 33) were classified as HPV positive, whereas LR genotypes (6 and 11) in addition to absent HPV were classified as HPV negative. Results were then categorized hierarchically according to a priori ordering of established cancer risk [3]: HPV 16 positive, else HPV 16 negative and HPV 18 positive, else HPV 16 and 18 negative and HPV 33 positive, else HPV 16 and 18 and 33 negative and HPV 31 positive, else HR-HPV negative including HPV 6 and 11. Based on this prior knowledge, we further merged HPV 16 with 18 and HPV 33 with 31, respectively.

Data were saved in a file with the specimen as a unit of registration. Within this file, each woman was given a pseudonymous number. SymPathy is the laboratory information system used in Norway for clinical pathology and cytology. Within this system, the HPV data were retrospectively linked to the correct cytology results.

### Follow-up

Until December 31th 2020 a single cohort, in which women were classified for their HPV status at baseline underwent passive follow-up through the NCCSP. Two hundred twenty-three women were HPV positive and 419 were HPV negative. Women with abnormal cytological findings had a cervical biopsy performed according to national guidelines. The Department of Clinical Pathology at UNN Tromsø receives and analyses all cervical cytology and cervical histology specimens from Tromsø and Finnmark counties. The existence of the National Cancer Register makes it possible to conduct studies with virtually no loss to follow-up. No national guidelines for HPV testing existed before 2005. The histopathological endpoint was defined as CIN3+ (CIN3, ACIS - adenocarcinoma in situ, or cancer). During the follow-up period, all incidents of CIN3+ were detected and compared with the HPV status at baseline.

### Statistical analyses

Statistical package SPSS, version 22.0, was used for the statistical analyses. The Chi-square test was used to determine whether there was a statistically significant difference within our cohort. Univariate analyses were done using the Kaplan-Meier method, and statistical significance between survival curves was assessed by the log rank test. To measure the uncertainty associated with the results we calculated the 95% confidence interval (CI). The Kaplan-Meier survival curves with 95% CI in the supplemental are computed using the survfit-function in R (version 4.0.3).

## Results

**Fig. 1:**
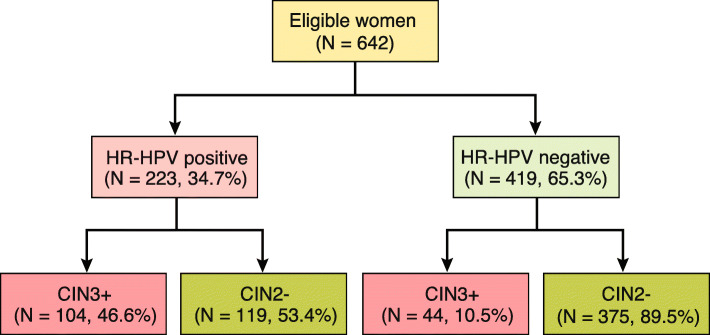
Flow chart illustrating the proportions of women developing CIN3+ following a positive and a negative baseline HPV test

Two hundred twenty-three women (34.7%) were HPV positive at baseline (Table 1, Additional file 1: Table S1 and Figure S1). During 30 years of follow-up, the detection rate of CIN3+ was 23.1% (148/642, 95% CI 19.9–26.5), (Table 1, Additional file 1: Table S1 and Figure S2). Of these, 70.3% (104/148, 95% CI 62.1–77.4) were HPV positive and 29.7% (44/148, 95% CI 22.6–37.9) were HPV negative at baseline. Figure 1 presents the proportions of women who developed CIN3+ following a positive and a negative test, 46.6% (104/223, 95% CI 40.0–53.4) and 10.5% (44/419 95% CI 7.8–13.9), respectively. Of the 642 patients, 148 women (23.1%) had HPV 16, and 33 (5.1%) had HPV 33 at baseline. In total, 48.6% (72/148) with HPV 16 and 57.6% (19/33) with HPV 33 developed CIN3+ (Additional file 1: Table S1). 53.4% (119/223, 95% CI 46.6–60.0) with HPV at baseline never developed CIN3+ during 30 years of follow-up. Table 1 presents HPV status at baseline and detection of CIN3+ in the different age groups during follow-up. Women aged 16–24 had the highest number of HPV at baseline, whereas the highest incidence of CIN3+ were seen in women aged 25–33. Thirty-six cases of CIN3+ were detected within the youngest age-group of whom 22 had CIN3+ at baseline. Overall, eight cases of cervical cancer were detected (Table 2). Five were HPV positive and all harboured HPV 16 at baseline. They developed cervical cancer three to 24 years after the positive baseline test. Four out of five had negative cytology at baseline. The remaining three cases of cervical cancer were present at baseline, all with a negative HPV test. All the three women had HSIL cytology at baseline (Table 2).

**Table 1:** HPV status at baseline and incidence of CIN3+ in the different age groups during 30 years of follow-up

Age (years)	Total	HPV positive at baseline (95% CI)	Histological confirmed CIN3+ (95% CI)
16–24	193	89 (46.1%, 39.0–53.4)	36 (18.7%, 13.6–25.0)
25–33	192	75 (39.1%, 32.2–46.4)	69 (35.9%, 29.2–43.2)
34–69	250	58 (23.2%, 18.2–29.0)	43 (17.2%, 12.9–22.6)
> 70	7	1	0 (0.0%)
Total	642	223 (34.7%, 31.1–38.6)	148 (23.1%, 19.9–26.5)

**Table 2:** Characteristics of cancer cases

Baseline			Follow-up		
Age	Cytology	HPV	Months to diagnosis	Age cancer diagnosis	Histology
24	Normal	16	37	27	SCC
26	Normal	16	50	30	SCC
29	HSIL	16	66	34	SCC
33	Normal	16	132	44	SCC
44	Normal	16	283	67	SCC
30	HSIL	Neg	0	30	SCC
32	HSIL	Neg	0	32	SCC
51	HSIL	Neg	0	51	ACC

**Fig. 2:**
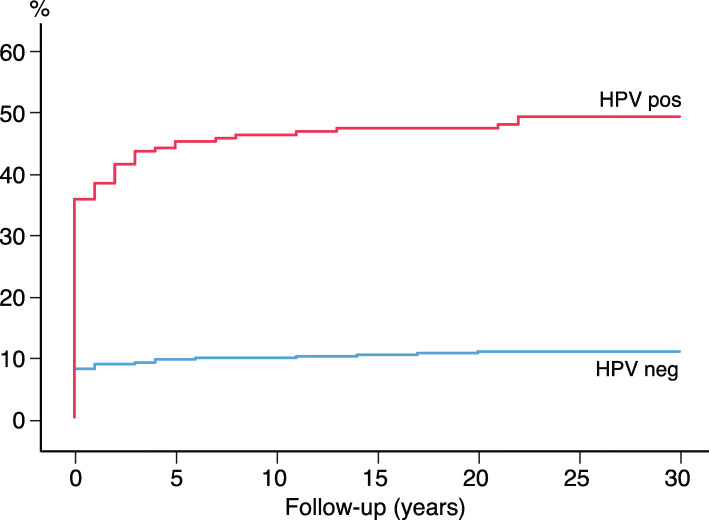
Cumulative incidence of CIN3+ by years of follow-up comparing women with a positive HPV test and a negative HPV test at baseline

**Fig. 3:**
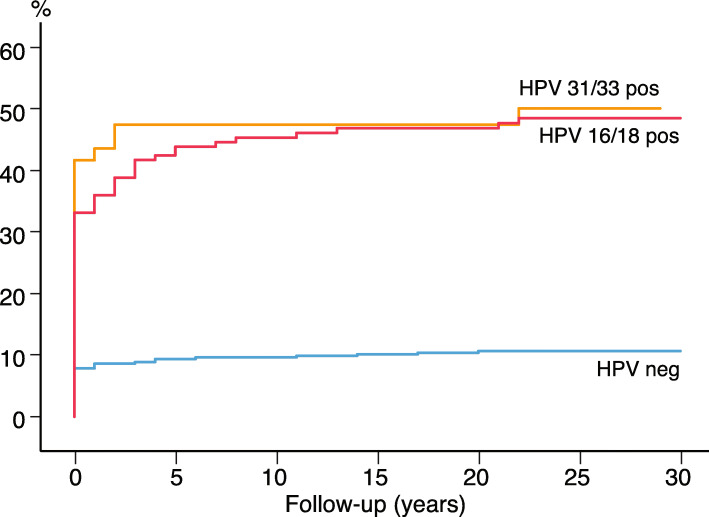
Cumulative incidence of CIN3+ by years of follow-up comparing women with HPV 31/33, HPV 16/18 and HPV negative

**Fig. 4:**
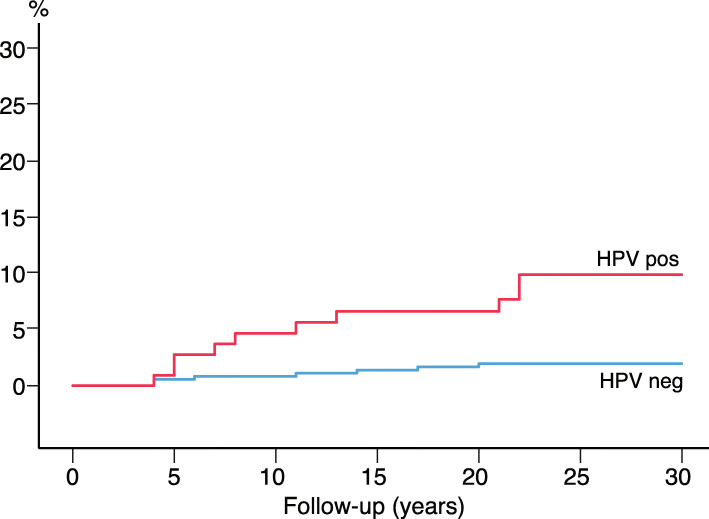
Cumulative incidence of CIN3+ by years of follow-up using 4 years quarantine from baseline, comparing women with a positive HPV test and women with a negative HPV test

**Fig. 5:**
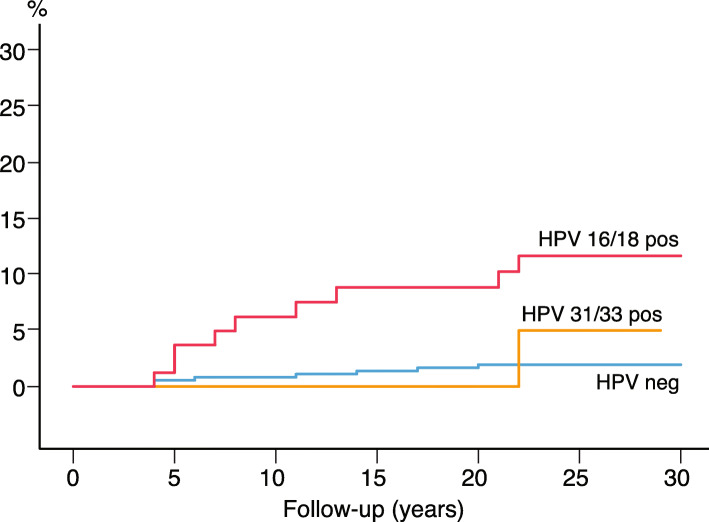
Cumulative incidence of CIN3+ by years of follow-up using 4 years quarantine from baseline. Comparing the women with HPV 16/18, HPV 31/33 and women with a negative HPV test

As illustrated in Fig. 2, most cases of CIN3+ were detected shortly after the baseline HPV test; 112 cases within the first year; 37.8% (56/148) with HPV 16, and 51.5% (17/33) with HPV 33 (data not shown). We further compared the HPV-negative, HPV 16/18 and HPV 31/33 positive women considering the incidence of CIN3+ (Fig. 3). The HPV 31/33 group had the highest incidence of CIN3+ at baseline. To predict the long-term risk of CIN3+ following the baseline HPV, all with CIN3+ present at baseline and first 3 years were ruled out. For the survival analysis based on a 4 years quarantine from baseline, 17 cases of CIN3+ were identified. Figure 4 shows the cumulative incidence of CIN3+ achieved by the baseline HPV positive and negative tests separately. The cumulative incidence of CIN3+ achieved by the baseline HPV 16/18 and HPV 31/33 is shown separately in Fig. 5. Figures 4 and 5 seem quite similar, indicating that beyond the quarantine there are few cases of CIN3+ due to HPV 31/33. The risk of CIN3+ in HPV 16/18-positive women remained high in a very long follow up: curves diverging even after 20 years. When merging HPV 16/33 using 4 years quarantine from baseline, HPV 18 and 31 does not contribute to the long-term risk (Additional file 1: Figure S3-S6). The cumulative incidence of CIN3+ after 4 years quarantine was almost similar for HPV 31/33 and HPV negative. Figures 2, 3, 4 and 5 is also found in supplemental with 95% CI (Additional file 1: Figure S7-S10).

Finally, the overall cumulative incidence of CIN3+ for each HPV genotype was analysed. HPV 33 shows a high risk of CIN3+ at baseline, but the long-term risk is mainly associated with HPV 16. This is further supported using 4 years of quarantine from baseline, having few cases of CIN3+ associated with other HPV types (Additional file 1: Figure S11, S12). Women with a negative HPV test had the same incidence of CIN3+ as those with LR-HPV genotypes, suggesting that detection of HPV 6 and 11 does not predict CIN3 + .

## Discussion

During 30 years of follow up, CIN3+ was detected in 23.1% (148/642) of the women in our cohort. The risk of CIN3+ among the HPV positive and HPV negative was 46.6 and 10.5%, respectively. Castle *et. al* estimated the absolute risk of CIN3+ in a US screening population to be 1.36% after 18 years of follow-up [18]. This notion suggests that our study population had a significantly higher risk of CIN3+, which is expected since they constitute a referral population. Women aged 25–33 years had the highest prevalence of CIN3+. Similar results were found in another recent study from 2019 [21]. Most cases of CIN3 did not progress to cervical cancer, presumably due to treatment or spontaneous regression. McCredie et al. compared the long-term risk of invasive cancer in women whose CIN3 lesion was not treated against those who had initial treatment followed by conventional management. The untreated were at high risk of cervical cancer in the follow-up, whereas the risk among the treated patients was very low. The cumulative incidence of invasive cervical cancer at 30 years in untreated and treated women with CIN3 was 31.3 and 0.7%, respectively [22]. A study by *Tjalma et.al* demonstrated that when the viral HPV is completely integrated, an L1 test looking for L1 expression only can miss this precancerous lesion, while the expression of oncoprotein E6 and E7 will remain present and will be captured by an E6/E7 test [23]. This may be a reason for the negative HPV tests in women with present cancer at baseline in our study.

The incidence of CIN3+ was significantly higher among the HPV positive, compared to the negative. The majority of CIN3+ was detected at, or within 3 years after, baseline. Using 4 years of quarantine from baseline, the incidence of CIN3+ was considerably lower. This is as expected because the first HPV infection often occurs soon after the first sexual intercourse and women with prevalent CIN3+ at baseline were treated. It is still likely that some women with a negative HPV test at baseline still acquired an HPV infection during 30 years of follow-up. The majority of CIN3+ followed a positive HPV test at baseline. More specifically, most of them had HPV 16. The long-term risk of CIN3+ was further quite similar for women with HPV 31 or 33 and those HPV negative. A study by Kjaer *et. al* discovered that HPV 16, 18, 31 and 33 infection, and especially HPV16 persistence, were associated with high absolute risks for progression to CIN3+. They also found that 2 years persistence of HPV16 carries a 50% risk of CIN3+ [4]. Current guidelines in the NCCSP recommend that women with a positive HPV have to be followed-up every 12 months until negative, or treatment-requiring CIN2+ is detected. After the HPV project at UNN was terminated in 1993, the HPV positive were followed up by cervical cytology only as recommended by the NCCSP, and there were no national guidelines for HPV testing before 2005. Women with a possibly persistent HPV-infection did not receive treatment if their cervical cytology was interpreted as normal. Unfortunately, some of these HPV positive women developed cervical cancer several years later that presumably could have been avoided if HPV tests were used in the follow-up. Four out of five women who developed cervical cancer after a positive HPV-test had negative cytology at baseline.

When removing the quarantine, women with HPV 33 and 16 had the highest incidence of CIN3+ (Additional file 1: Table S1 and Figure S3-S6, S11-S12). This is consistent with another study which found that HPV 16 and 33 appeared to have a higher oncogenic potential than other HPV types [24]. Tjalma et al. found that the most common HPV types in women with high-grade CIN were 16/33/31, and in invasive cervical cancer 16/18/45, supporting that HPV 33 carries a high risk of CIN3+ even though the risk of cancer is considerably lower than HPV 18 [7]. Different HPV types have different oncogenic potential, but it is not clear how many HPV types a screening test should contain. Most HPV DNA tests detect 13 or 14 HPV types. Our test detected four HR-HPV types, including HPV 16, 18, 31 and 33 in addition to LR-HPV 6 and 11, causing 78.3% of all cervical cancer [3]. In a Norwegian study using a five-type HPV mRNA test (16, 18, 31, 33 and 45), the six-year cumulative risk of CIN3+ for the HPV mRNA positive and the HPV mRNA negative was 19.7 and 0.62%, respectively [25]. Sundström et al. discovered that HPV types 16, 18, 31, 33, 45 or 52 was found in 689 of 808 screen-detected Invasive Cervical Cancers (85.3%) and that addition of eight more HR-HPV types increased prevalence by only 12 of 808 cases (1.5%, for all these eight types together) [26]. They suggest that screening limited to the six most oncogenic HPV genotypes could greatly improve program specificity. The nonavalent HPV vaccine (9vHPV) protects against the seven most important HPV-viruses (16, 18, 45, 33, 31, 52, 58), causing 90% of cervical cancer. It is important to consider the trade-off between sensitivity and specificity of the diagnostic test when designing screening algorithms.

The strengths of this study include the long follow-up time: curves for HPV 16/18 diverging even after 20 years. All women had a colposcopic examination done at baseline, and follow-up was ensured by the call-recall system initiated by the NCCSP. Another strength is the virtual absence of loss to follow-up because of the existence of the National Cancer Register. This study also has some limitations. Participant had no second colposcopic examination unless cytology findings suggested so, leading to several women being followed-up by cytology only. Because we measured HPV only once, we have no information about persistent infection and its role in the risk of progression to high-grade cervical lesions. Due to a small number of events, it is difficult to conclude on the long-term risk for other HPV than HPV 16.

## Conclusion

HPV status at baseline is predictive for the subsequent risk of developing CIN3+. Women with a positive HPV test in 1990–1992 had a significantly higher risk of CIN3+ during 30 years of follow-up compared to those with a negative test. HPV 16 provided the greatest long-term risk of cervical cancer. All patients with HPV at baseline should be followed up until negative.

## Supplementary Information

**Additional file 1 **: **Table S1**. HPV genotypes at baseline, cases of CIN3+ and PPV CIN3+ during follow-up. PPV = positive predictive value. **Figure S1**: HPV status at baseline by age. **Figure S2**. Cases of CIN3+ during follow-up by age. **Figure S3**. Cumulative incidence of CIN3+ by years of followup comparing HPV 16/33, HPV 18/31 and HPV negative. **Figure S4**. Cumulative insidence of CIN3+ by years of follow-up comparing HPV 16/33, HPV 18/31 and HPV negative, with 95% CI. **Figure S5**. Cumulative incidence of CIN3+ using 4 years quarantine from baseline, comparing women with HPV 16/33, HPV 18/31 and HPV negative. **Figure S6**. Cumulative incidence of CIN3+ by years of follow up using 4 years quarantine from baseline, comparing HPV 16/33, HPV 18/31 and HPV negative, with 95% CI. **Figure S7**. Cumulative incidence of CIN3+ by years of follow-up comparing women with a positive HPV test and a negative HPV test at baseline, with 95% CI. **Figure S8**. Cumulative incidence of CIN3+ by years of follow-up comparing women with HPV 31/33, HPV 16/18 and HPV negative, with 95% CI. **Figure S9**. Cumulative incidence of CIN3+ by years of follow-up using 4 years quarantine from baseline, comparing women with a positive HPV test and women with a negative HPV test, with 95% CI. **Figure S10**. Cumulative incidence of CIN3+ by years of follow-up using 4 years quarantine from baseline, comparing women with HPV 16/18, HPV 31/33 and women with a negative HPV test, with 95% CI. **Figure S11**. Cumulative incidence of CIN3+ by years of follow-up comparing all HPV genotypes. **Figure S12**. Cumulative incidence of CIN3+ by years of follow-up using 4 years quarantine from baseline, comparing all HPV genotypes.

## Data Availability

The dataset used and analysed during the current study is available from the corresponding author on reasonable request.

## Abbreviations

ACC: Adenoid Cystic Carcinoma
CI: Confidence Interval
CIN: Cervical intraepithelial neoplasia
DNA: Deoxyribonucleic acid
HPV: Human Papilloma Virus
HR-HPV: High-risk Human Papilloma Virus
ICC: Invasive Cervical Cancer
LR-HPV: Low-risk Human Papilloma Virus
mRNA: Messenger Ribonucleic Acid
NCCSP: Norwegian Cancer Screening Program
PCR: Polymerase chain reaction
REK: Regional Ethics Committee
SCC: Squamous Cell Carcinoma
SPSS: Statistical Package for Social Sciences
UNN: University Hospital of Northern Norway
