# Supplementary material for: Risk of cervical intraepithelial neoplasia grade 3 or higher (CIN3+) among women with HPV-test in 1990–1992, a 30-year follow-up study
Source: Infect Agent Cancer. 2021 Jun 22;16:46. doi: 10.1186/s13027-021-00386-z (PMC8220730; doi:10.1186/s13027-021-00386-z)
Supplement: Supplementary file 1 — Additional file 1 : Table S1. HPV genotypes at baseline, cases of CIN3+ and PPV CIN3+ during follow-up. PPV = positive predictive value. Figure S1: HPV status at baseline by age. Figure S2. Cases of CIN3+ during follow-up by age. Figure S3. Cumulative incidence of CIN3+ by years of followup comparing HPV 16/33, HPV 18/31 and HPV negative. Figure S4. Cumulative insidence of CIN3+ by years of follow-up comparing HPV 16/33, HPV 18/31 and HPV negative, with 95% CI. Figure S5. Cumulative incidence of CIN3+ using 4 years quarantine from baseline, comparing women with HPV 16/33, HPV 18/31 and HPV negative. Figure S6. Cumulative incidence of CIN3+ by years of follow up using 4 years quarantine from baseline, comparing HPV 16/33, HPV 18/31 and HPV negative, with 95% CI. Figure S7. Cumulative incidence of CIN3+ by years of follow-up comparing women with a positive HPV test and a negative HPV test at baseline, with 95% CI. Figure S8. Cumulative incidence of CIN3+ by years of follow-up comparing women with HPV 31/33, HPV 16/18 and HPV negative, with 95% CI. Figure S9. Cumulative incidence of CIN3+ by years of follow-up using 4 years quarantine from baseline, comparing women with a positive HPV test and women with a negative HPV test, with 95% CI. Figure S10. Cumulative incidence of CIN3+ by years of follow-up using 4 years quarantine from baseline, comparing women with HPV 16/18, HPV 31/33 and women with a negative HPV test, with 95% CI. Figure S11. Cumulative incidence of CIN3+ by years of follow-up comparing all HPV genotypes. Figure S12. Cumulative incidence of CIN3+ by years of follow-up using 4 years quarantine from baseline, comparing all HPV genotypes. [file 13027_2021_386_MOESM1_ESM.pdf]

Tables

HPV type	HPV baseline	Cases of CIN3+	PPV CIN3+ % (95% CI)
Negative	379	41	10.8 (8.0-14.5)
6	23	2	8.7 (1.5-29.5)
11	17	1	5.9 (0.3-30.8)
16	148	72	48.6 (40.4-57.0)
18	15	3	20.0 (5.3-48.6)
31	27	10	37.0 (20.1-57.3)
33	33	19	57.6 (39.4-74.1)
Total	642	148	23.1 (19.9-26.5)

Table S1: HPV genotypes at baseline, cases of CIN3+ and PPV CIN3+ during follow-up. PPV = positive predictive value

Figures

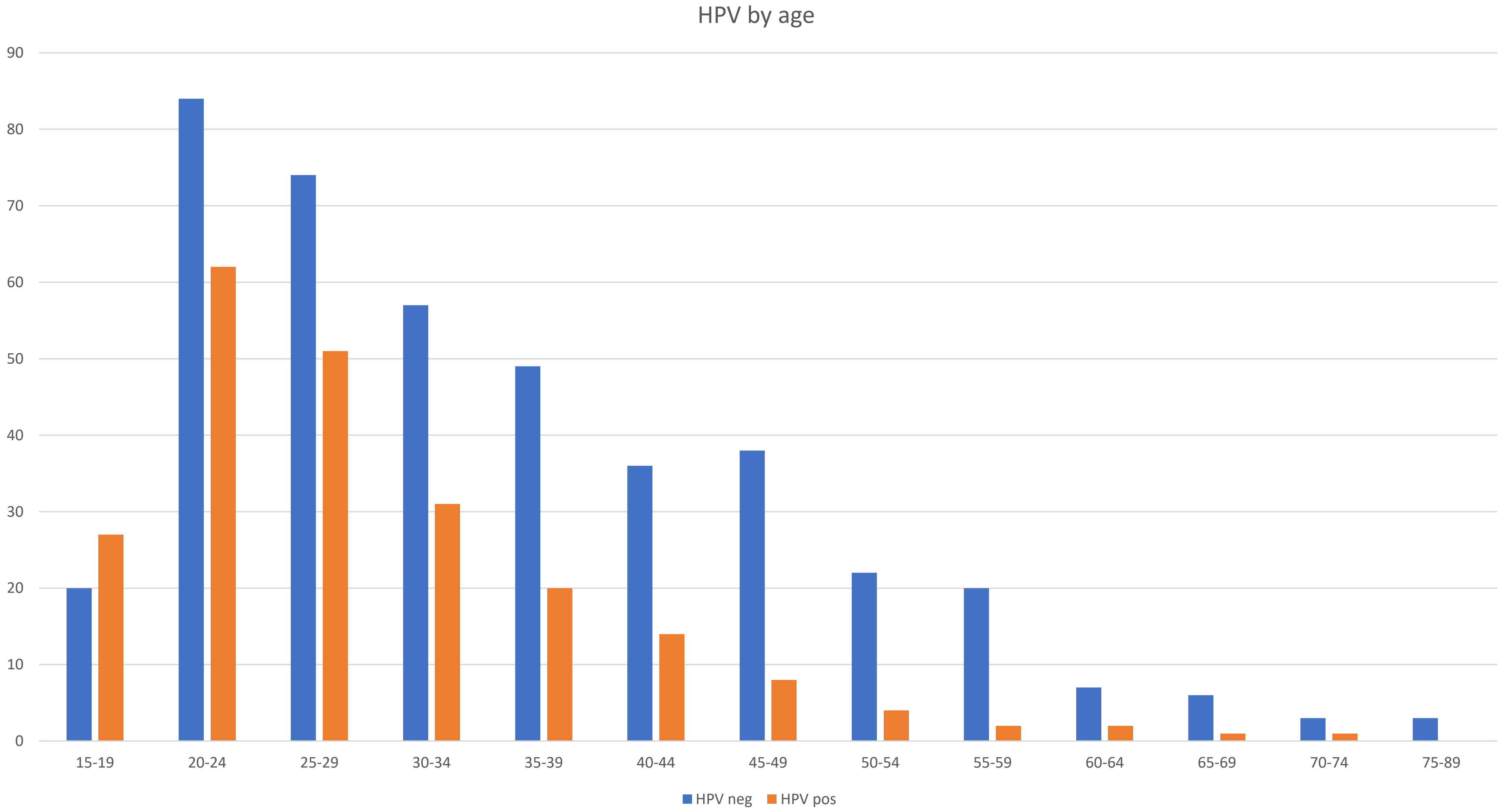

Figure S1: HPV status at baseline by age.

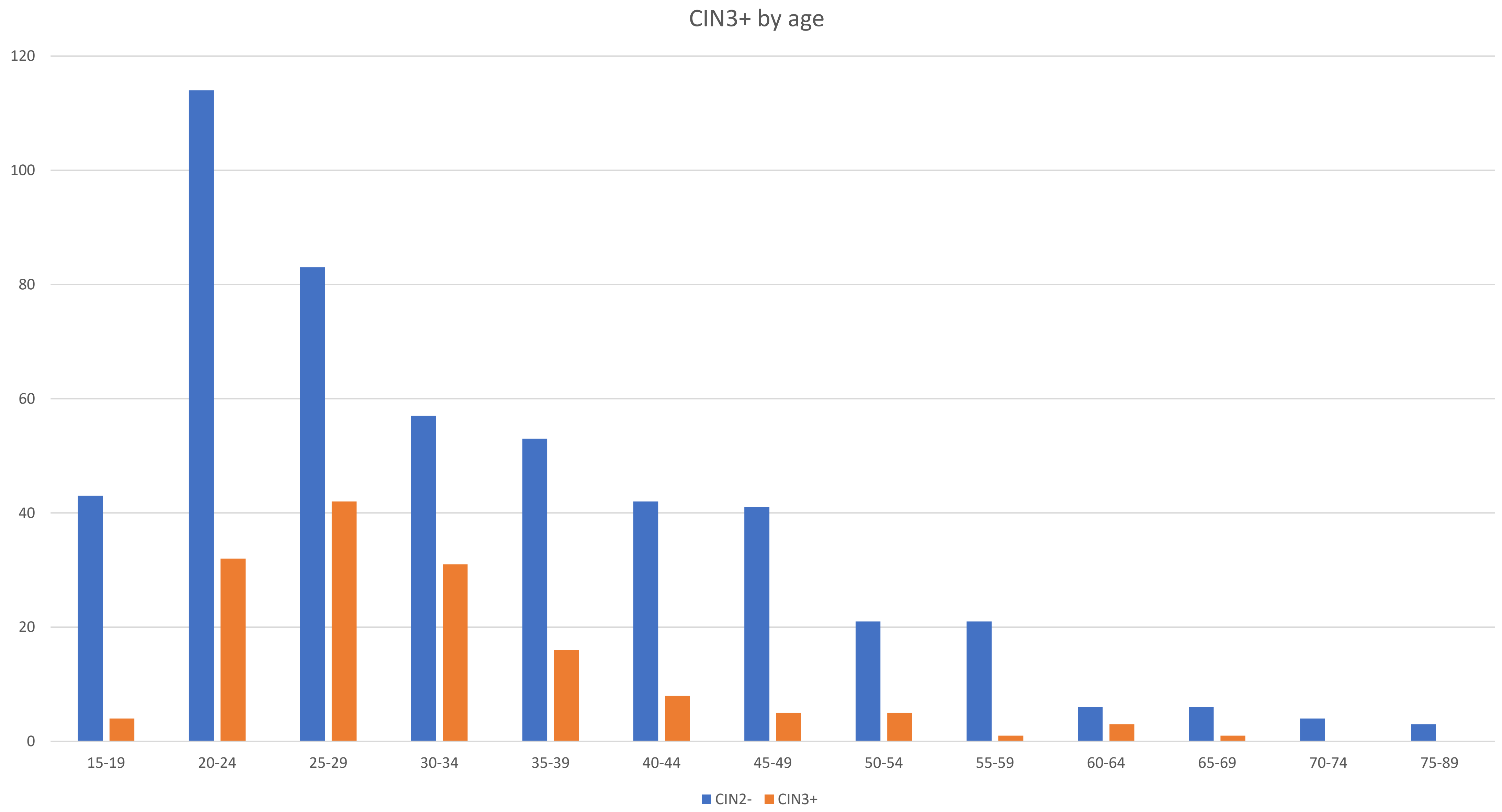

Figure S2: Cases of CIN3+ during follow-up by age.

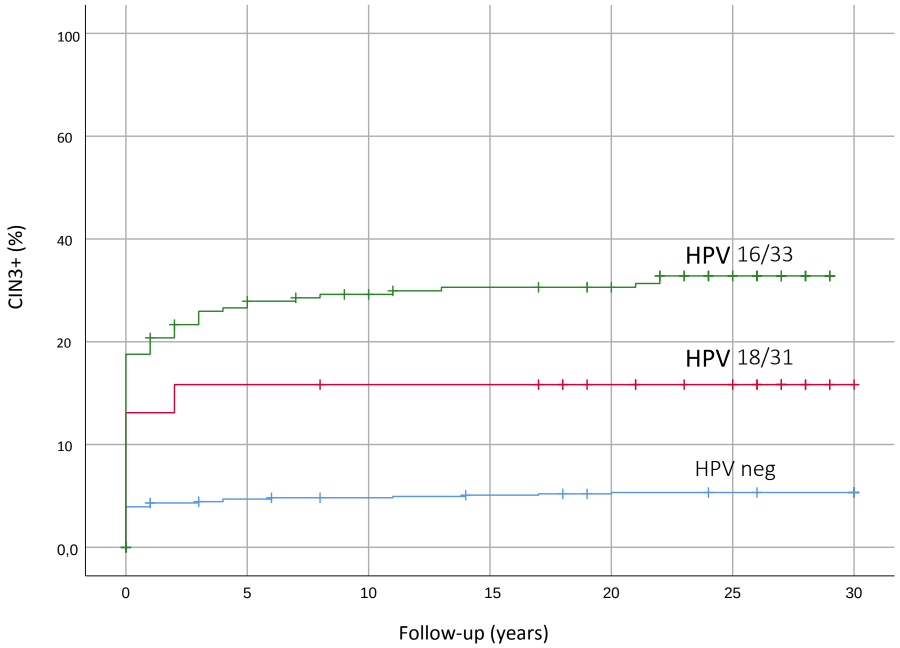

Figure S3: Cumulative incidence of CIN3+ by years of follow-up comparing HPV 16/33, HPV 18/31 and HPV negative.

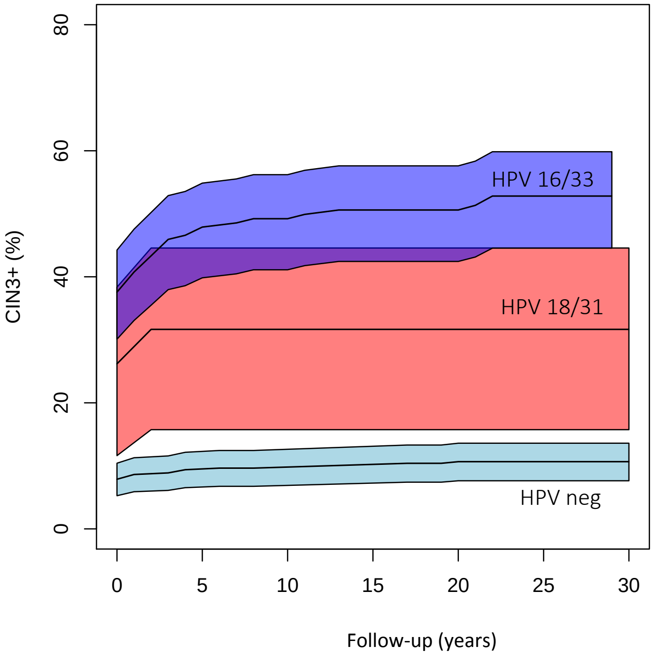

Figure S4: Cumulative insidence of CIN3+ by years of follow-up comparing HPV 16/33, HPV 18/31 and HPV negative, with 95% CI.

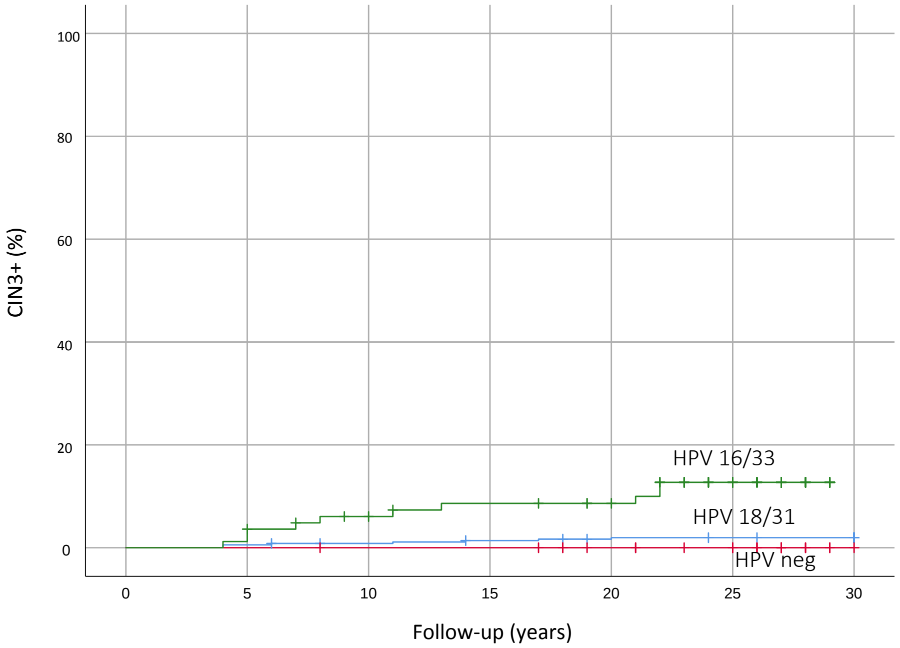

Figure S5: Cumulative incidence of CIN3+ using four years quarantine from baseline, comparing women with HPV 16/33, HPV 18/31 and HPV negative.

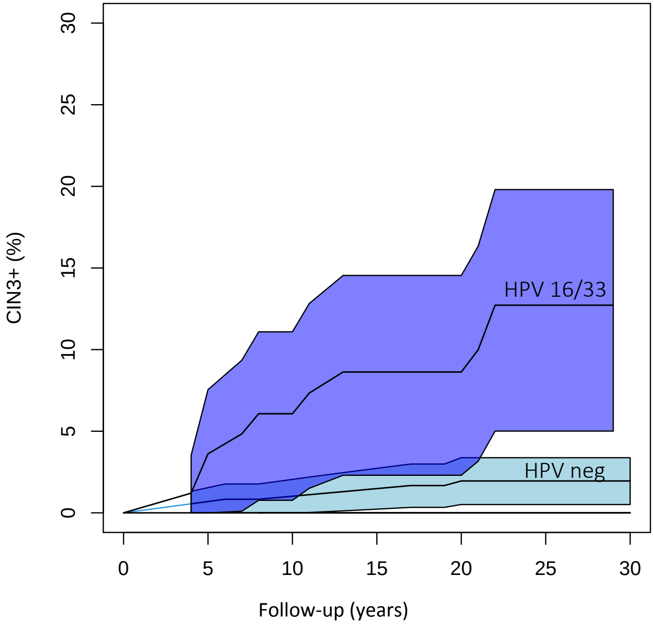

Figure S6: Cumulative incidence of CIN3+ by years of follow up using four years quarantine from baseline, comparing HPV 16/33, HPV 18/31 and HPV negative, with 95% CI.

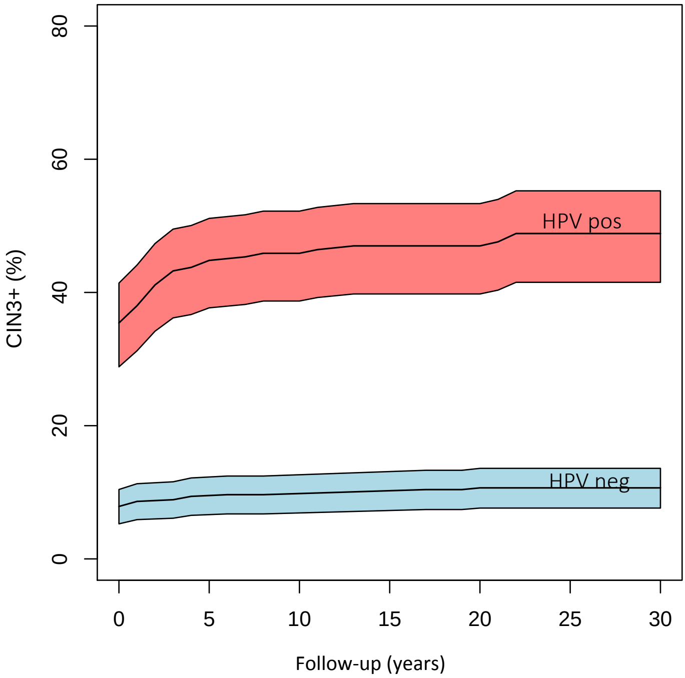

Figure S7: Cumulative incidence of CIN3+ by years of follow-up comparing women with a positive HPV test and a negative HPV test at baseline, with 95% CI.

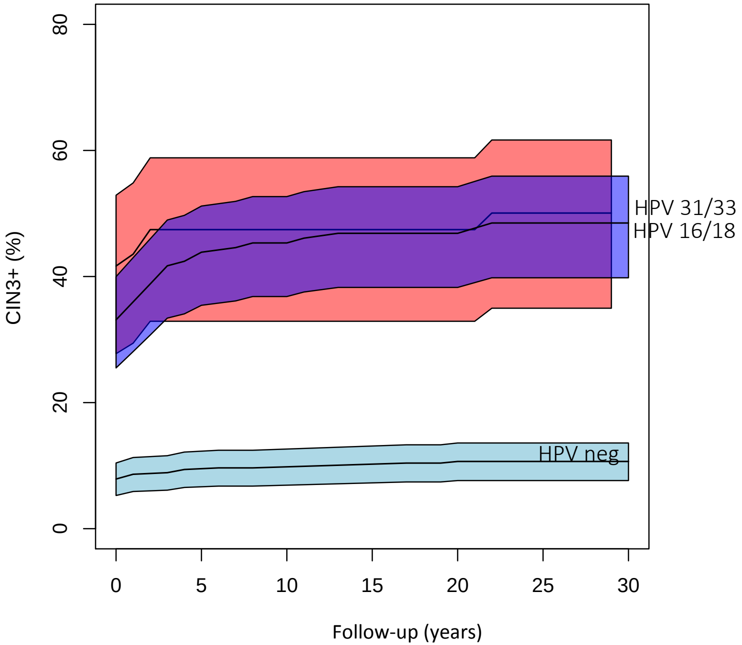

Figure S8: Cumulative incidence of CIN3+ by years of follow-up comparing women with HPV 31/33, HPV 16/18 and HPV negative, with 95% CI.

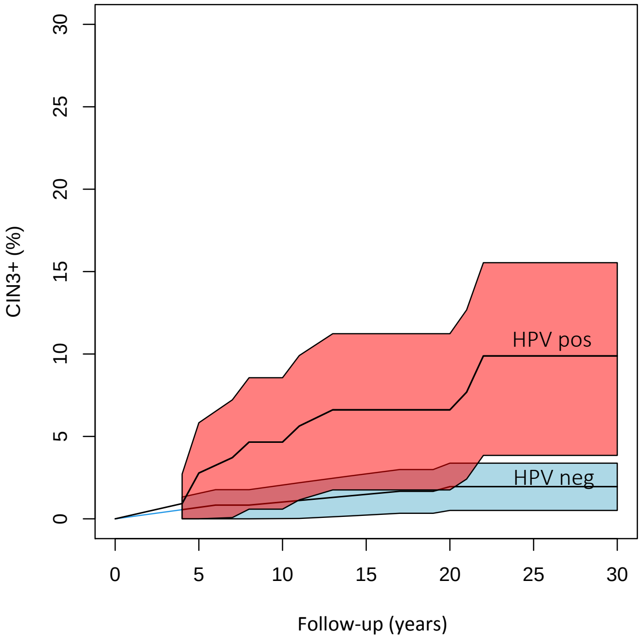

Figure S9: Cumulative incidence of CIN3+ by years of follow-up using four years quarantine from baseline, comparing women with a positive HPV test and women with a negative HPV test, with 95% CI.

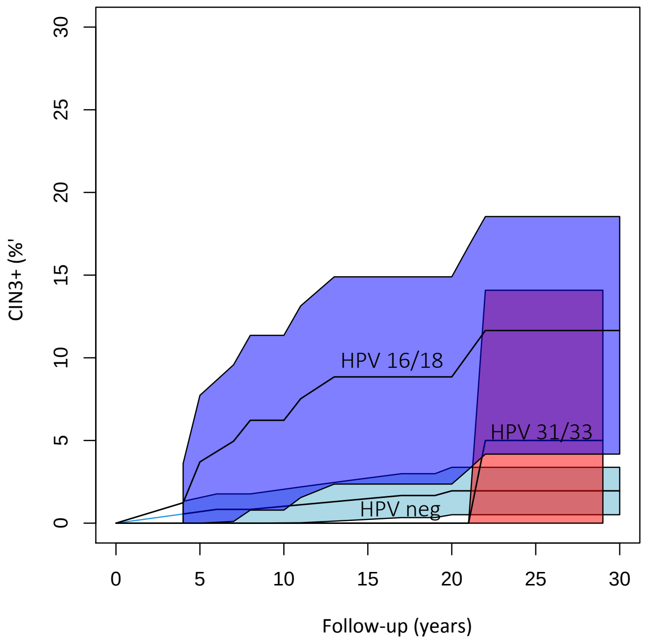

Figure S10: Cumulative incidence of CIN3+ by years of follow-up using four years quarantine from baseline, comparing women with HPV 16/18, HPV 31/33 and women with a negative HPV test, with 95% CI.

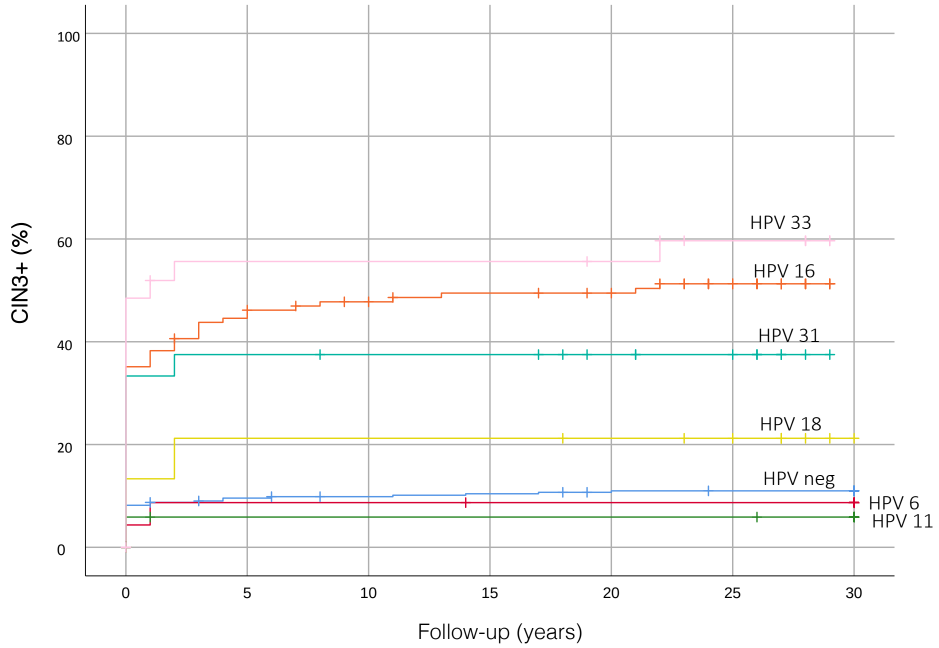

Figure S11: Cumulative incidence of CIN3+ by years of follow-up comparing all HPV genotypes.

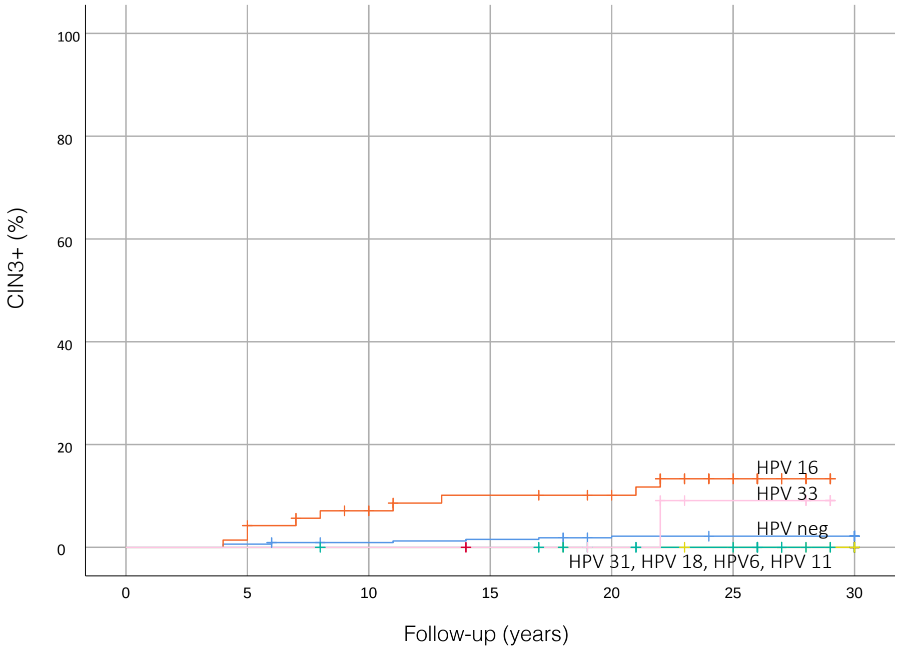

Figure S12: Cumulative incidence of CIN3+ by years of follow-up using four years quarantine from baseline, comparing all HPV genotypes.
